# Low-Temperature Synthesis of Titanium Oxynitride Nanoparticles

**DOI:** 10.3390/nano11040847

**Published:** 2021-03-26

**Authors:** Felicitas Jansen, Andreas Hoffmann, Johanna Henkel, Khosrow Rahimi, Tobias Caumanns, Alexander J. C. Kuehne

**Affiliations:** 1 Institute of Organic and Macromolecular Chemistry, Ulm University, Albert-Einstein-Allee 11, 89081 Ulm, Germany; felicitas-1.jansen@uni-ulm.de (F.J.); andreas.hoffmann@uni-ulm.de (A.H.); 2 DWI—Leibniz Institute for Interactive Materials, Forckenbeckstraße 50, 52076 Aachen, Germany; johanna.henkel@rwth-aachen.de (J.H.); rahimi@dwi.rwth-aachen.de (K.R.); 3 GFE Central Facility for Electron Microscopy, RWTH Aachen University, 52074 Aachen, Germany; caumanns@gfe.rwth-aachen.de

**Keywords:** metal oxynitride, nanoparticles, colloid synthesis, energy storage, capacitor

## Abstract

The synthesis of transition metal oxynitrides is complicated by extreme reaction conditions such as high temperatures and/or high pressures. Here, we show an unprecedented solution-based synthesis of narrowly dispersed titanium oxynitride nanoparticles of cubic shape and average size of 65 nm. Their synthesis is performed by using titanium tetrafluoride and lithium nitride as precursors alongside trioctylphosphine oxide (TOPO) and cetrimonium bromide (CTAB) as stabilizers at temperatures as low as 250 °C. The obtained nanoparticles are characterized in terms of their shape and optical properties, as well as their crystalline rock-salt structure, as confirmed by XRD and HRTEM analysis. We also determine the composition and nitrogen content of the synthesized particles using XPS and EELS. Finally, we investigate the applicability of our titanium oxynitride nanoparticles by compounding them into carbon fiber electrodes to showcase their applicability in energy storage devices. Electrodes with titanium oxynitride nanoparticles exhibit increased capacity compared to the pure carbon material.

## 1. Introduction

Transition metal oxynitrides, for example titanium oxynitride (TiON) nanoparticles, represent powerful active sites when added to photocatalytic devices, or to electrodes for capacitors, batteries, and fuel cells [1,2,3]. Typically, TiON nanoparticles are prepared via a solvothermal route, which involves annealing the respective oxide with ammonia at temperatures between 600 °C and 850 °C [2,4,5,6,7,8,9]. Alternative routes require high pressures or complex reaction conditions, such as sol–gel preparation, laser pyrolysis, and plasma-supported atomic layer deposition [2,3,9,10,11,12,13,14,15]. Transition metal oxynitrides combine properties of both the pure metal oxides and the pure metal nitrides. While oxynitrides share good thermal and chemical stability with the corresponding metal oxide and metal nitride, oxides usually exhibit poor electrical conductivity but high capacitance [9,16,17,18]. By contrast, transition metal nitrides show a good electrical conductivity and fairly low capacitance [9,16,19,20]. Therefore, TiON and other transition metal oxynitrides are explored as additives in electrode or capacitor materials [5,21,22,23,24]. When immobilized on carbon nanotubes, the TiON composite materials can be used as electrodes for supercapacitors, where the capacity increases by a factor of 5 versus pristine carbon nanotube electrodes and by a factor of 2 compared to titanium oxide carbon/nanotube composites [9]. This example shows that TiON nanoparticles are capable of boosting the surface area as well as the conductivity, producing high-performance electrodes for energy storage applications [10,23,25,26,27,28].

Unfortunately, due to the synthetic hurdles, these examples are limited to small-scale devices, with little chance of being transferred to large-scale industrial applications. A route towards narrowly dispersed nanoparticles of titanium group oxynitrides synthesized at low temperatures in solution could enable an upscaling of the process while reducing the cost and complexity of the reactor. Furthermore, solution synthesis could facilitate more complex particles such as core–shell architectures. The use of a suitable stabilizer could enable uniform and individually stabilized nanoparticles within the resulting dispersion. This colloidal stability would offer easy handling, formulation, and compounding for versatile applications.

Here, we develop a low-temperature solution-based synthesis yielding polycrystalline titanium oxynitride nanoparticles of a uniform, cubic shape at temperatures as low as 250 °C. We characterize the obtained particles and show their applicability and performance by employing them in carbon-fiber-based capacitor devices.

## 2. Results and Discussion

**Figure 1:**
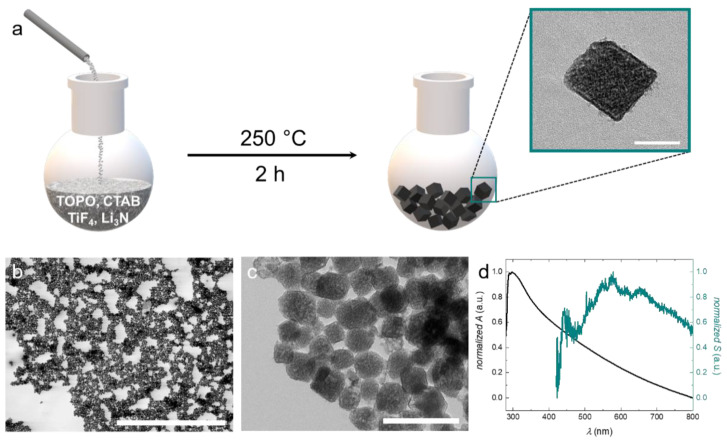
(**a**) Schematic illustration of the reaction procedure. TiF_4_ and Li_3_N are dissolved in trioctylphosphine oxide (TOPO) and cetrimonium bromide (CTAB) and reacted at 250 °C for 2 h to form cubic or cube-like titanium oxynitride (TiON) nanoparticles. (**b**) STEM image of the narrowly dispersed TiON nanoparticles. (**c**) TEM image of the synthesized TiON nanoparticles. (**d**) Optical properties of the TiON nanoparticles. The black curve displays the absorbance of the TiON nanoparticles; the cyan curve displays the contribution of scattering to the absorbance. The scale bars represent: (**a**) = 50 nm, (**b**) = 5 μm, (**c**) = 200 nm.

As reagents, we apply titanium tetrafluoride TiF_4_ as the titanium source and Li_3_N as the nitrogen source to obtain titanium oxynitride nanoparticles (Figure 1a). Oxygen is supplied by the atmosphere. A similar approach was previously published for tantalum nitride nanoparticles, where a higher reaction temperature and different stabilizers were applied [29]. We adapt the procedure and optimize the reaction conditions to obtain TiON nanoparticles.

The reaction is carried out in solution under a reduced oxygen atmosphere (N_2_ with ~5% O_2_) at 250 °C for 2 h. This low reaction temperature enables the use of standard laboratory equipment such as glassware reactors, and oil baths or heating mantles. As a high-boiling solvent, we use trioctylphosphine oxide (TOPO), which also serves as a stabilizer for the particles upon their formation. Generally, in solution-based syntheses, particle formation is based on two processes: nucleation and growth. With respect to the resulting size, particle growth is the decisive step. It is known that this process can be significantly influenced by the choice of stabilizers and surfactants [30]. Appropriate stabilizers confer colloidal stability and lead to the formation of small particles with narrow size distribution. However, if colloidal stability is insufficient during growth, the nuclei will aggregate and thus larger particles with a broad size distribution will be obtained. Besides TOPO, we use cetrimonium bromide (CTAB) as a supplemental surfactant because, like TOPO, it is known as a structure- and shape-directing agent for the synthesis of nanoparticles [31,32,33,34]. Analysis of the particles using scanning transmission electron microscopy (STEM) gives insight into the narrowly dispersed particle size distribution (see Figure 1b). For determining the mean particle size, we average over 200 particles from several STEM and transmission electron microscopy (TEM) images. The particles exhibit a cubic or cube-like shape with an average size of 65 nm (see inset Figure 1a,c). The particle size distribution of the synthesized TiON nanoparticles is displayed in Figure S1.

The contrast variation of the particles in TEM analysis indicates that they are polycrystalline. Between the particles, organic residues of low contrast can be observed (see Figure 1c). These organic residues most likely represent layers of stabilizer that form around the particles during synthesis. We move on to characterize the optical properties of our particles, which can provide information about the crystal structure and O/N ratio in oxynitride nanoparticles. We first investigate the scattering of the nanoparticles by dark-field microscopy. The scattering spectrum exhibits a broad peak with maxima between 600 nm and 700 nm. Pure titanium nitride (TiN) nanoparticles with a size between 56 nm and 70 nm exhibit maximal scattering intensities around 700 nm [35].

Therefore, the scattering properties of our synthesized TiON particles correspond well to those of the pure nitride as well as the pure oxide, as described in the literature [36]. By contrast, the absorbance exhibits a maximum just above 300 nm, which represents the contribution of absorption of TiO_2_ and not TiN [37]. Since this analysis is inconclusive with regard to the crystal structure and the nitrogen content, we first turn to crystal structure analysis.

**Figure 2:**
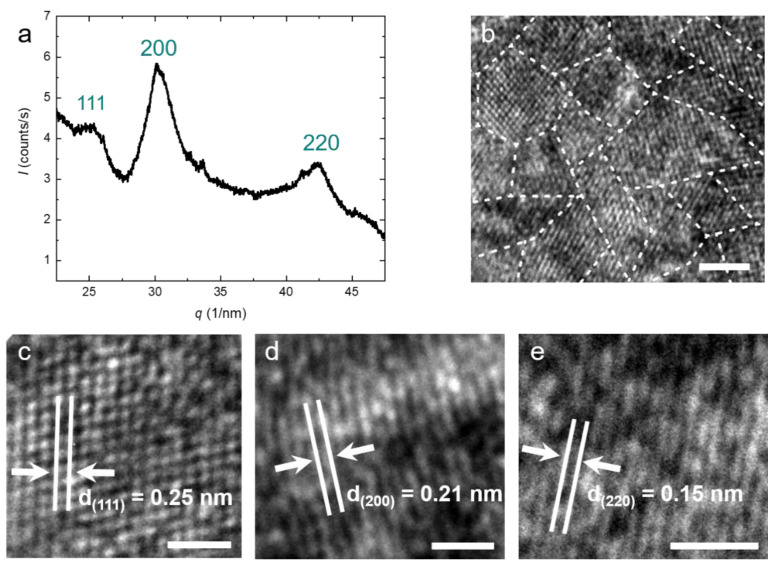
(**a**) XRD spectrum of the purified TiON nanoparticles. The peak position relative to the fundamental q* is obtained as √3:2:√8, which indicates the face-centered cubic (fcc) crystal structure for the TiON nanoparticles with a lattice parameter *a* = 0.416 nm; (**b**) HRTEM image of the synthesized TiON nanoparticles. The image shows the crystalline domains; (**c**–**e**) HRTEM images of the interatomic layer distance. The measured distances correspond to the peaks observed in the XRD spectrum (**a**). The scale bars represent (**b**) = 5 nm, (**c**–**e**) = 1 nm.

Generally, TiON nanoparticles can occur in anatase, rock-salt, rutile, and pseudobrookite crystal structures, depending on the nitrogen content and the reaction conditions [38,39,40,41]. X-ray diffraction (XRD) of our particles shows three main peaks corresponding to the (111), (200), and (220) planes (see Figure 2a). The peak positions indicate a face-centered cubic (fcc) crystal structure for the particles with a lattice parameter of *a* = *b* = *c* = 0.416 nm. This is in agreement with typical lattice parameters for TiON in the literature. The XRD peaks are relatively broad, indicating polycrystallinity. We determine the average domain size using Scherrer’s equation and find *D* = 2.9 nm [38]. This confirms our previous hypothesis that the particles are polycrystalline. Both the polycrystallinity as well as the average domain size are confirmed by high-resolution TEM (HRTEM) (see Figure 2b). Depending on the orientation of the crystallites, the spacing between two atomic layers varies (see Figure 2b). This distance directly correlates with the peaks in the XRD spectrum [19,42]. All distances belonging to the three peaks in the XRD can also be found in HRTEM images, confirming the rock-salt crystal structure (Figure 2c–e).

**Figure 3:**
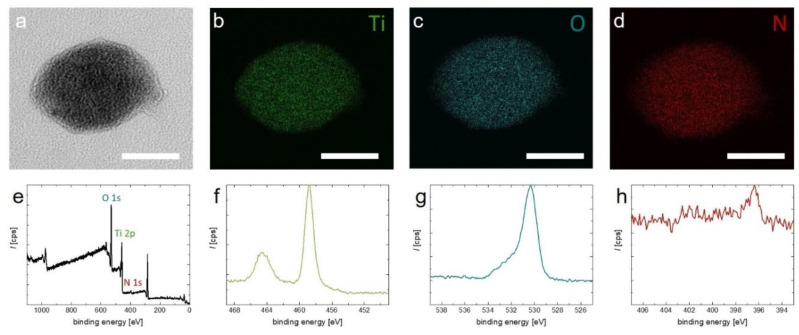
(**a**–**d**) EELS mapping of a TiON nanoparticle. (**a**) TEM image of the analyzed particle. (**b**–**d**) Elemental mapping of titanium, oxygen, and nitrogen, respectively; (**e**–**h**) XPS spectra of the synthesized TiON nanoparticles. Image (**e**) shows a full scan; images (**f**–**h**) show the spectra regions of Ti 2p, O 1s, and N 1s, respectively. The scale bars in (**a**–**d**) = 40 nm.

Electron energy loss spectroscopy (EELS) mapping of an individual TiON nanoparticle verifies that the particles contain titanium and oxygen as well as nitrogen (see Figure 3a–d). EELS also allows determination of the ratio of O to N, which is found to be 5 to 1, respectively (see Figure S2). XPS confirms the presence of nitrogen (see Figure 3e–h). In the full scan, the peaks belonging to Ti 2p, O 1s, and N 1s are clearly present. This is confirmed when zooming in to the regions of the respective elements (Figure 3f–h). The relatively weak nitrogen signal in XPS is explained by limited penetration depth of X-rays and an oxide passivation layer that forms on top of the particles (see Figure 3a).

The synthesis of the TiON nanoparticles can also be extended to zirconium and hafnium oxynitride nanoparticles, but with reduced nitrogen content compared to our TiON nanoparticles (see Figure S3).

**Figure 4:**
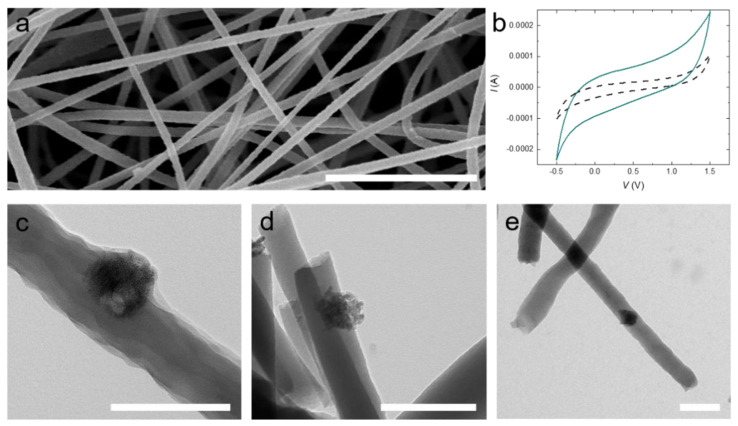
(**a**) SEM image of electrospun polyacrylonitrile (PAN) nanofibers containing TiON nanoparticles; (**b**) Cyclic voltammograms of uncompounded carbon-fiber-nonwovens (CFNs) (black dashed line) and CFNs containing 1 wt% TiON nanoparticles (cyan line) at a scan rate of 0.005 V/s; (**c**) TEM image of electrospun PAN nanofibers containing TiON nanoparticles; (**d**) TEM image of electrospun stabilized PAN nanofibers containing TiON nanoparticles; (**e**) TEM image of electrospun carbonized PAN nanofibers containing TiON nanoparticles. The scale bars in (**a**) = 2 µm, (**c**–**e**) = 200 nm.

TiON nanoparticles present promising nanoadditives for electrode materials due to their conductivity, chemical resistance, and high surface area [23,24,25,26,27,28]. To test our TiON nanoparticles, we incorporate them into a carbon-fiber-nonwoven (CFN). We compound the TiON nanoparticles with a polyacrylonitrile (PAN) solution (1 wt% with respect to PAN) as a precursor for the CFN, which we process by electrospinning (see Figure 4a). The nanofibers are converted to carbon fibers by thermal stabilization and carbonization at 550 °C. Incorporation of TiON nanoparticles into the electrospun PAN nanofibers is confirmed by TEM and cyclic voltammetry (see Figure 4b–e).

The TiON nanoparticles are clearly discernible from the carbon-based nanofiber. Most of the particles sit at the fiber interface, allowing contact with the electrolyte when in an electrode setup, for example in a battery or supercapacitor (see Figure 4c–e). We conclude from the rectangular shape of the voltammograms in Figure 4b that no faradaic reaction is present. However, the (double-layer) capacitance of the CFN composite electrode containing 1 wt% TiON is increased by a factor of seven compared to pristine CFN (uncompounded CFN: 50 mF/g; TiON compounded CFN: 373 mF/g). We speculate that this increase in capacitance is attributable to the improved conductivity and the increased surface area of the TiON composite CFN electrode.

## 3. Conclusions

In summary, we developed a low-temperature synthesis process for TiON nanoparticles. The obtained particles were narrowly dispersed and exhibited an average size of 65 nm. We used several analytical methods to determine their structure and exact composition. The TiON nanoparticles exhibited a rock-salt crystal structure and consisted of titanium, oxygen, and nitrogen, the latter two mentioned with a ratio of 5 to 1, respectively. The synthesis route shows potential to be transferred to other transition metals such as zirconium and hafnium. Besides improving the electronic properties in carbon-based electrode materials, TiON nanoparticles show EPR activity (see Figure S4), making them interesting candidates for spintronic applications. Further investigations into the magnetic properties of the particles are required to assess all potential applications of these interesting materials that can be accessed using low-temperature syntheses.

## 4. Materials and Methods

All chemicals and solvents are used without further purification. The following chemicals are purchased from Sigma Aldrich: cetrimonium bromide (CTAB, 99%), lithium nitride (Li_3_N, 100%), titanium (IV) fluoride (TiF_4_), trioctylphosphine oxide (TOPO, 99%), and zirconium (IV) fluoride (ZrF_4_, 99.9%). Methanol (analytical reagent grade) is obtained from Fisher Chemical. Hafnium (IV) fluoride (HfF_4_, 99%) is purchased from abcr (abcr GmbH, Karlsruhe, Germany).

### 4.1. Synthesis

*Titanium oxynitride (TiON) nanoparticles (NPs):* TOPO (3.0 g, 7.76 mmol), CTAB (1.4 g, 3.84 mmol), titanium (IV) fluoride (0.15 g, 1.21 mmol), and lithium nitride (0.16 g, 4.59 mmol) are mixed under nitrogen (with an oxygen content of approximately 3–5%) and heated to 250 °C. After keeping this temperature for 2 h the mixture is cooled to 70 °C. Methanol (25 mL) is added to the dark grey dispersion, leading to the flocculation of a black precipitate. Purification is performed in 2 mL batches. The sample is centrifuged (10 min, 6000 rpm) and the supernatant is decanted, followed by redispersion in distilled water by sonication for 5 min at 80 °C. For further purification, the following cycle is repeated 10 times: centrifugation (2 min, 6000 rpm), removal of the supernatant, and redispersion in distilled water by sonication (5 min at 80 °C). The titanium oxynitride NPs are obtained as a black powder after drying (yield = 183 mg).

*Zirconium oxynitride (ZrON) NPs:* The synthesis and purification of ZrON NPs is performed analogously to the TiON synthesis described above using the following chemicals: TOPO (3.0 g, 7.76 mmol), CTAB (1.0 g, 2.74 mmol), zirconium (IV) fluoride (0.20 g, 1.20 mmol), and lithium nitride (0.16 g, 4.59 mmol). The reaction mixture is a brown dispersion, leading to the flocculation of a dark gray precipitate. During purification, the centrifuge is set to 13,400 rpm. The ZrON NPs are obtained as a gray powder (yield > 34 mg).

*Hafnium oxynitride (HfON) NPs:* The synthesis and purification of HfON NPs is performed analogously to the TiON synthesis described above using the following chemicals: TOPO (3.0 g, 7.76 mmol), CTAB (1.2 g, 3.29 mmol), hafnium (IV) fluoride (0.31 g, 1.22 mmol), and lithium nitride (0.16 g, 4.59 mmol). The reaction mixture is a brown dispersion, leading to the flocculation of a dark gray precipitate. During purification the centrifuge is set to 13,400 rpm. The HfON NPs are obtained as a light gray powder (yield > 42 mg).

*Carbon-fiber-titanium oxynitride composite:* TiON NPs are dispersed in DMF by sonication for 10 min at 80 °C. The resulting dispersion is added to a 12 wt% PAN-DMF solution to obtain a 8 wt% PAN solution with 1 wt% TiON nanoparticles (to PAN). This mixture is electrospun on ITO-covered glass slides over the course of 1 h. (Voltage: 17 kV; tip-to-substrate distance: 15 cm; pump rate: 0.5 mL/h.) The obtained nonwoven is stabilized at 250 °C with a heating rate of 5 °C/min in air atmosphere and further carbonized at 550 °C (to avoid melting of the glass slides) with a heating rate of 5 K/min for 60 min in inert gas atmosphere.

### 4.2. Analytical Methods

*Scanning transmission electron microscopy (STEM):* A Hitachi UHR-FESEM SU9000 (Tokyo, Japan) is used for scanning transmission electron microscopy. The particles are dispersed in methanol and the sample is put on a carbon TEM grid using the drop-on-grid procedure.

*Transmission electron microscopy (TEM):* The TEM measurements are carried out with a Zeiss Libra 120 (Oberkochen, Germany) microscope operating at 120 kV. The particles are dispersed in methanol and the sample is put on a silicon monoxide TEM grid using the drop-on-grid procedure.

*High-resolution transmission electron microscopy (HRTEM):* HRTEM measurements are performed with a FEI Tecnai F20 (Hillsboro, OR, USA) operating at 200 kV. The particles are dispersed in methanol and the sample is deposited on a lacey carbon film grid via drop-on-grid.

*Electron energy loss spectroscopy (EELS):* For EELS mapping, a Zeiss Libra 120 microscope operating (Oberkochen, Germany) at 120 kV is used. The EEL spectra to determine the oxygen/nitrogen ratio are recorded by TEM with a HAADF (High-angle annular dark-field imaging) detector.

*X-ray diffraction (XRD):* XRD measurements are performed on a Malvern Empyrean X-ray diffractometer (Worcestershire, UK) by PANalytical. Cu Kα radiation with a wavelength of *λ* = 1.5418740 Å is applied.

*UV/Vis spectroscopy:* A Jasco V-780 Spectrophotometer (Pfungstadt, Germany) is used for UV/Vis spectroscopy. The measurements are carried out between 350 and 1000 nm with a scan rate of 400 nm/min. The sample is diluted in distilled water and filtered through a PTFE syringe filter (1 μm) before measurement.

*Dark-field microscopy:* For dark-field microscopy, a Shamrock SR-303i-B Spectrometer (Belfast, UK) with an iDus 420 CCD detector from Andor Technology is used. An objective lens of type UPLFLN60XOI from Olympus is applied. The integration period is chosen to be between 10 and 30 s.

*X-ray photoelectron spectroscopy (XPS):* The XPS spectra are recorded with an aluminum anode (1486.6 eV) using the Kratos AXIS ultra XPS system (Manchester, UK).

*Cyclic voltammetry:* For electrochemical characterization, a potentiostat/galvanostat (PGSTAT302N-Metrohm GmbH, Herisau, Switzerland) is used. The CFN electrodes are contacted through the ITO layer on a glass substrate in a three-electrode setup with a Ag wire as a pseudo-reference electrode and a Pt disc as a counter electrode. A 0.1 M LiClO_4_ solution in EMIM TFSI is used as the electrolyte. The capacitance *C* is determined by the following equation:

$$C\;=\;\frac{\int_{V_{1}}^{V_{2}}I\;\left(V\right)dV}{2\;\times \;v\left(V_{2}-V_{1}\right)}$$

with *I* as current, *V*_1_ and *V*_2_ as the voltage limits, and *v* as the scan rate.

*Electron paramagnetic resonance (EPR):* The spectra are recorded using a Bruker ELEXSYS E580 X-band EPR system (Karlsruhe, Germany) with an ER 4122 SHQE cavity, using the software xEPR for data acquisition. The measurement is performed at microwave frequency f X ≈ 9.84 GHz, with a resonance quality factor Q = 8000, a power 0.15 mW (30 dB attenuation in xEPR). Modulation amplitude and frequencies are 1 G/100 KHz. Prior to the measurement, samples are inserted into 4 mm quartz EPR tubes (Wilmad 707-SQ-100M, Vineland, NJ, USA). The acquisition time for each spectrum is 40 s. The signal corresponds to: g = 2.0026 ± 0.0002.

## Data Availability

Data is contained within the article and Supplementary Material.

## Supplementary Materials

The following are available online at https://www.mdpi.com/article/10.3390/nano11040847/s1. Figure S1. Particle size distribution of the synthesized TiON nanoparticles. Figure S2. EELS spectrum of a TiON nanoparticle. Figure S3. (a)–(c) Characterization of the synthesized ZrON and HfON nanoparticles by TEM and XRD. Figure S4. EPR spectrum for the synthesized TiON, ZrON, and HfON nanoparticles.
