# Supplementary material for: Low-Temperature Synthesis of Titanium Oxynitride Nanoparticles
Source: Nanomaterials (Basel). 2021 Mar 26;11(4):847. doi: 10.3390/nano11040847 (PMC8065472; doi:10.3390/nano11040847)
Supplement: Supplementary file 1 [file nanomaterials-11-00847-s001.pdf]

Supplementary Materials

Low-Temperature Synthesis of Titanium Oxynitride Nanoparticles

Felicitas Jansen[1,2], Andreas Hoffmann[1], Johanna Henkel[2], Khosrow Rahimi[2], Tobias Caumanns[3], Alexander J. C. Kuehne[1,2,*]

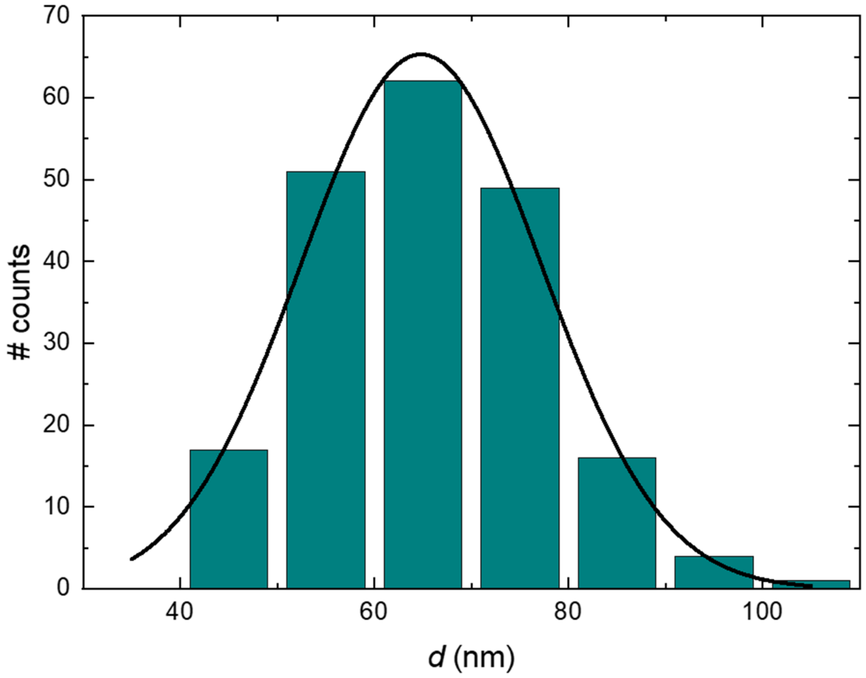

Figure S1. Particle size distribution of the synthesized titanium oxynitride (TiON) nanoparticles (NPs).

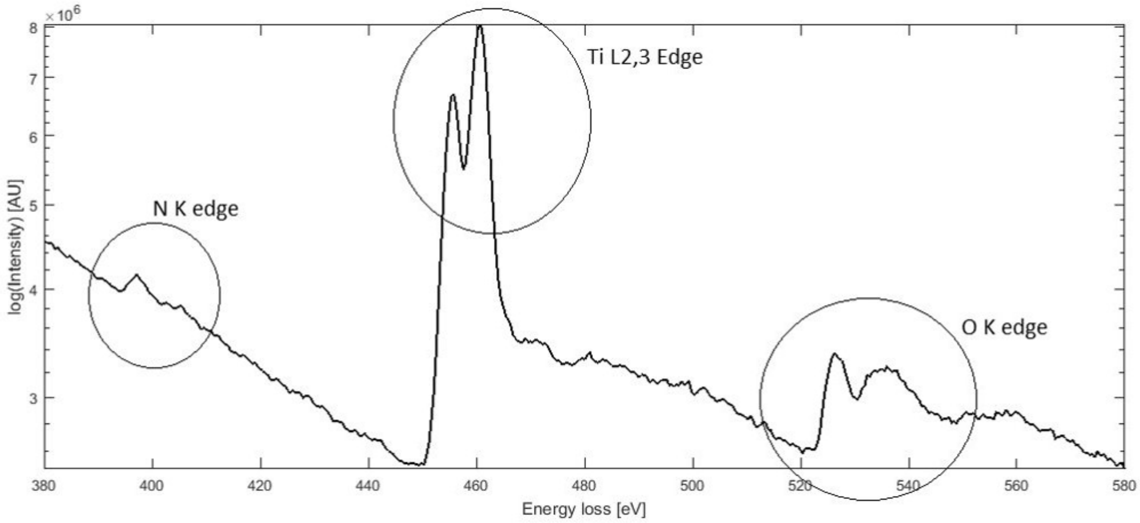

Figure S2. EEL spectrum of a TiON NP. The ratio of oxygen to nitrogen is 5 to 1.

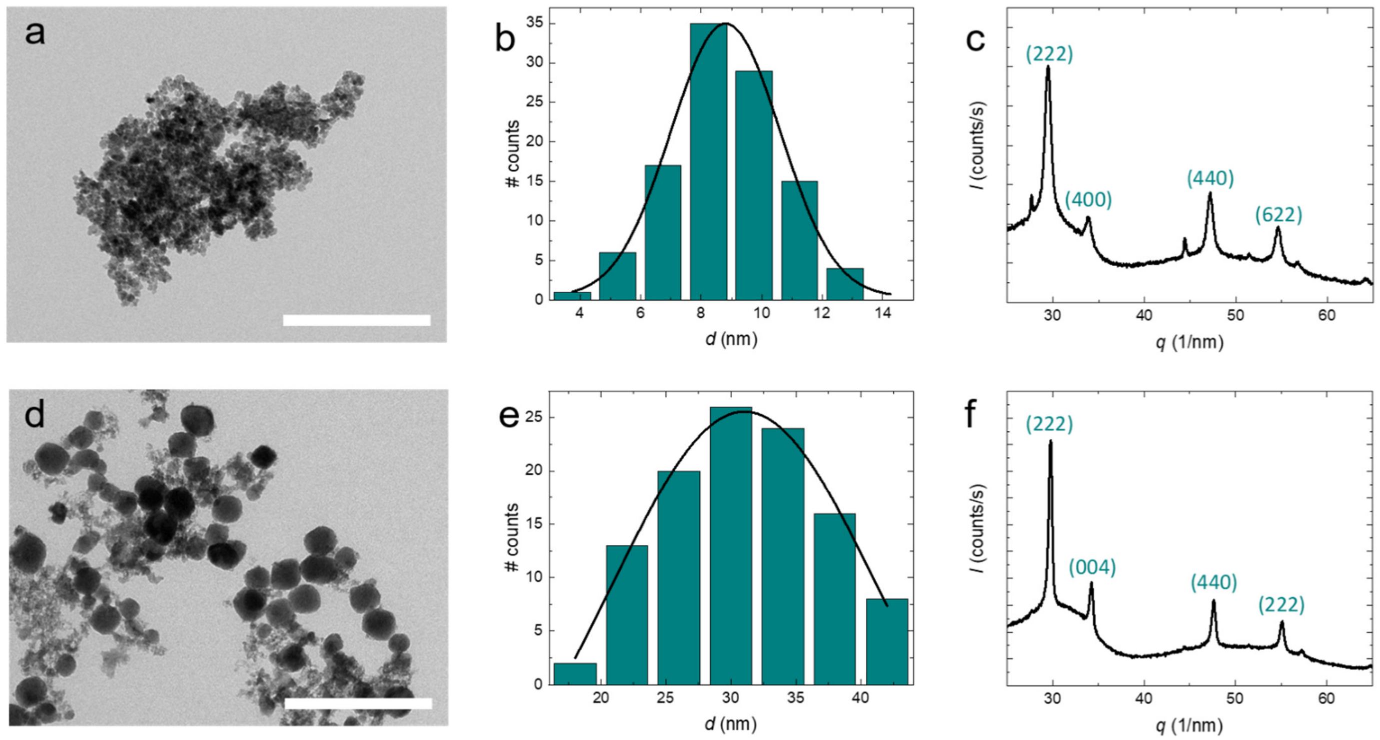

Figure S3. (**a**–**c**) Characterization of the synthesized ZrON NPs. (**a**) TEM image, the scale bar represents 200 nm; (**b**) Particle size distribution; (**c**) XRD spectrum. (**d**–**f**) Characterization of the synthesized HfON NPs. (**d**) TEM image, the scale bar represents 200 nm; (**e**) Particle size distribution; (**f**) XRD spectrum.

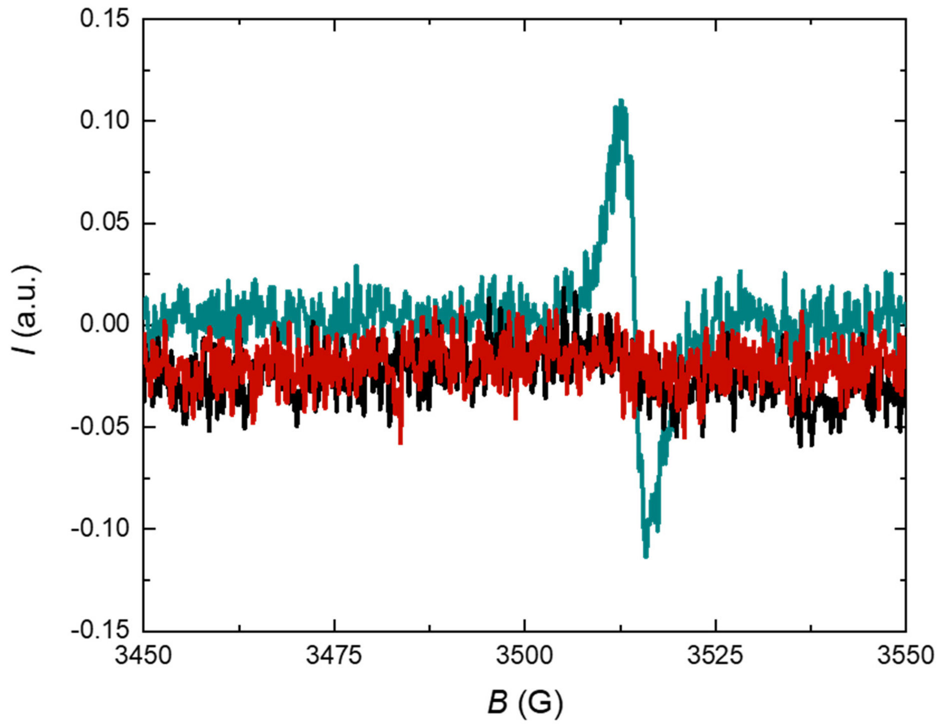

Figure S4. EPR spectrum for the synthesized TiON (cyan), ZrON (black), and HfON (red) NPs.
